# Recent practical researches in the development of gluten-free breads

**DOI:** 10.1038/s41538-019-0040-1

**Published:** 2019-05-01

**Authors:** Hiroyuki Yano

**Affiliations:** Food Research Institute, National Agriculture and Food Research Organization, Tsukuba, Ibaraki 305-8642 Japan

**Keywords:** Technology, Nutrition

## Abstract

Wheat bread is consumed globally and has played a critical role in the story of civilization since the development of agriculture. While the aroma and flavor of this staple food continue to delight and satisfy most people, some individuals have a specific allergy to wheat or a genetic disposition to celiac disease. To improve the quality of life of these patients from a dietary standpoint, food-processing researchers have been seeking to develop high-quality gluten-free bread. As the quality of wheat breads depends largely on the viscoelastic properties of gluten, various ingredients have been employed to simulate its effects, such as hydrocolloids, transglutaminase, and proteases. Recent attempts have included the use of redox regulation as well as particle-stabilized foam. In this short review, we introduce the ongoing advancements in the development of gluten-free bread, by our laboratory as well as others, focusing mainly on rice-based breads. The social and scientific contexts of these efforts are also mentioned.

## Introduction

The aroma emanating from a bread bakery is unmistakably alluring. The flavor and crunchy texture of wheat breads sharpen our appetite and satisfy our basic human cravings for comfort as well as nutrition. Indeed, human beings are so enchanted by bread that it is much more than a “staple food”; it has been called “the staff of life”. Breadmaking has a long and fascinating story.^1–4^ It is generally accepted that breadmaking dates back to the New Stone Age, from 8000 to 10,000 BC, and originated around the Fertile Crescent and consisted of emmer and einkorn wheat grains.^1^ At first the grains were consumed as porridge. Then, grains that had been hand-crushed using knocking stones were mixed with water and baked on a heated stone with a cover of hot ash, resulting in an unfermented, flat bread. Later, around 6000 BC, people in southern Mesopotamia started using sourdough,^5^ speculated to have been developed accidently in an abandoned mixture of flour and water. This first leavened bread dough, which contained fermentation gas, swelled up in the baking process. In ~3000 BC, the Egyptians improved bread by adding yeast, developing what would become the prototype of modern bread. They dehulled and milled wheat grains using saddle querns, the most ancient type of quern stones,^6^ which were later replaced by rotary querns and are used even today. Breadmaking and beer production in Egypt are closely related and are considered evidence of a high degree of civilization.^7^ Bread was made not only with flour prepared from raw grains, but sometimes also with malt (germinated grains). Moreover, water with a blend of cooked and uncooked malt was used in brewing. The mixture was strained free of husk before inoculation with yeast.

The precise origin of bread has still not been determined. Recent reports show archaeobotanical evidence that the origins of bread date back to 14,400 years ago.^8^ Progress in archaeology will eventually clarify the origin of bread, along with some sense of how bread fits into the larger culture of ancient civilizations. Wheat bread is now one of the most representative food in the world. A unique property of wheat gluten realizes bread with high quality. However, some genetically predisposed people cannot eat wheat bread, because gluten causes harmful reactions to them. In this short review, we will summarize the gluten-dependent swelling mechanism of wheat bread and the recent scientific effort to make bread without gluten.

## Modern wheat breadmaking

Simply stated, breadmaking is composed of three steps: mixing/sheeting, fermenting, and baking processes.^9^ In the mixing process, wheat flour, water, yeast, sugar, salt, oil, and other components are mixed and kneaded. Here, the ingredients are blended homogeneously and hydrated, resulting in the development of the all-important gluten network.^10^ Gluten is made from two major wheat proteins together comprising 85% of wheat endosperm protein: gliadin and glutenin. Kneading of wheat dough promotes the hydrogen bonding and disulfide cross-linking interactions of these proteins, eventually producing a viscoelastic and highly conformational protein network termed “gluten”.^11^ Yeast grows fast in the dough, feeding on supplemental sugar, until it consumes all available oxygen. Then, it shifts metabolism from aerobic respiration to anaerobic fermentation. In the subsequent fermentation process, yeast generates fermentation gas, mainly composed of carbon dioxide and other components, such as ethanol:

1 $${\mathrm{C}}_6{\mathrm{H}}_{12}{\mathrm{O}}_6 \to 2{\mathrm{C}}_2{\mathrm{H}}_5{\mathrm{OH}} + 2{\mathrm{CO}}_2$$

**Fig. 1:**
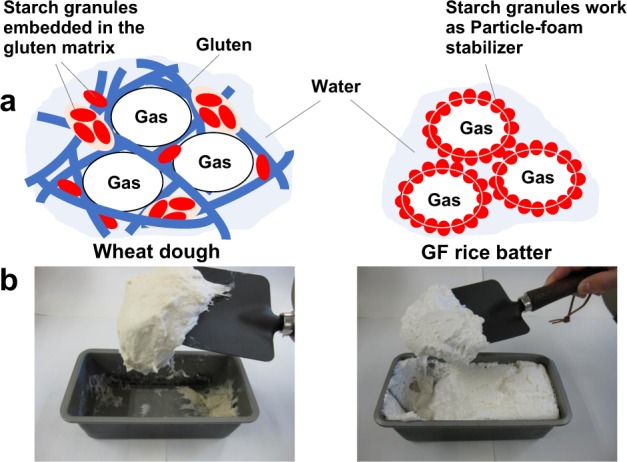
Comparison of the swelling mechanism (**a**) and appearance (**b**) of fermenting wheat dough and additive-free, gluten-free (GF) rice batter

In wheat dough, the gas is confined in the continuous “gluten matrix”,^12^ which is composed of the viscoelastic gluten network and other components, such as starch granules and water (Fig. 1a). Thus, in the beginning of the fermentation process, many small gas cells are produced throughout the dough, like so many small balloons. As the fermentation proceeds, each small gas cell grows bigger, and the dough rises. In the following baking process, the gas cell inflates further by heat, resulting in the expansion, namely, “oven spring” of the dough.^13^ The starch molecules are gelatinized by heat, so that the gluten matrix forming the envelopes of the “balloons” become hardened, thus constructing the stable crumb framework.^14^ Concurrently, the crust, or surface of the bread dough, is hardened as well as browned by the Maillard reaction between the sugars and amino acids.^15^ Finally, the breadmaking is completed, emitting a fresh aroma.^16^

The preparation of ingredients, especially flour, is also a critical step. Wheat grain is composed mainly of three parts: the endosperm, germ, and bran.^17^ In the endosperm, which is the major constituent of the polished grain, starch granules are embedded in a protein matrix.^18^ Wheat flour is produced by grinding whole-wheat grains or polished ones mechanically.^19^ Impact mills, such as hammer mills and pin mills, accomplish particle size reduction by exposing seeds to a set of rotating hammer or pins that fracture the seeds, while roller and stone mills compress the seeds between two hardened surfaces.^20^ During the milling of wheat grains, a portion of the starch granules are mechanically damaged.^21^ The extent of the damage depends on wheat variety (hard or soft type) as well as milling conditions. In the mixing and fermentation steps of breadmaking, damaged starch accelerates the absorption of water to the starch granules, resulting in the activation of local amylases, leading to the degradation of starch molecules into dextrin and maltose.^22^ Consequently, yeast activity and the final bread volume is increased. However, excessive starch damage produces wet or sticky dough and bread with poor quality. Thus, control of flour quality in terms of the starch damage is critical in the milling industry.^23^

In other words, intact and damaged starch granules each have their respective role in the making of wheat bread—and, as we will show, in rice-flour breads as well. In the case of wheat dough, intact starch granules constitute the gluten matrix, while damaged ones activate fermentation. Generally, the extent of starch damage in commercially available wheat flours is 10–15%.^19^

## Social demand for gluten-free food

### Gluten intolerance

While the unique viscoelastic property of gluten realizes wheat bread with high quality, some people choose to or must follow a gluten-free diet. Recent reviews well summarize the background and status quo of gluten-free diets,^24,25^ so only the outline will be mentioned here. Gluten intolerance includes autoimmune celiac disease (CD), wheat allergy, and non-celiac gluten sensitivity (NCGS). Celiac disease is an autoimmune disorder caused by genetic as well as environmental factors.^26^ In CD patients, ingestion of gluten leads to small intestinal damage, typically leading to malabsorption. Its prevalence in the United States and Europe is estimated to reach about 1%. Gluten protein has protease-resistant regions in its structure.^27^ Digestion of gluten in the human gastrointestinal tract generates “pathogenic” peptides that occasionally reach the lamina propria, where the peptides are deamidated by local transglutaminase.^28^ The modified gluten peptides have a higher affinity to human leukocyte antigen (HLA)–DQ2 as well as HLA–DQ8 molecules,^29^ which are present only in the small percentage of people carrying the HLA–DQ2 or the DQ8 haplotype.^30^ This bonding results in the presentation of the gluten peptides to T cells, thereby triggering further malignant immune response in those with CD. In addition, tissue transglutaminase cross-links covalently to gliadin molecules. The protein complexes with new epitopes are considered to trigger the primary immune response as well. Antibodies against tissue transglutaminase are characteristic of CD.^31^

In contrast, food allergy to wheat is characterized by T helper type 2 (Th2) activation, which can result in immunoglobulin E (IgE) and non-IgE-mediated reactions.^32^ The IgE-mediated wheat allergy reactions usually occur immediately after contact of wheat, and are characterized by the occurrence of wheat-specific IgE antibodies in serum. Ingestion of wheat causes food allergy, while inhalation of wheat causes respiratory allergy to genetically predisposed individuals. A food allergy to wheat may cause a life-threatening reaction, such as anaphylaxis and wheat-dependent, exercise-induced anaphylaxis.^33^ In contrast, repetitive exposure to wheat flour may cause baker’s asthma or rhinitis, mostly characterized as occupational allergic diseases.^34^ Non-IgE- mediated food allergy reactions to wheat usually occur hours or even days after ingestion of wheat products and are characterized by chronic eosinophilic inflammation of the gastrointestinal tract.^35^ There is a variability among reports of wheat allergy prevalence due to the differences in the diagnostic criteria, methodology, age, and geography.^36^ The prevalence of wheat allergy is estimated to be 0.9% in the United Kingdom (based on questionnaire response),^37^ 3.6% in the United States (based on measurement of anti-wheat-specific IgE antibodies),^38^ and 0.2% in Japan (based on a combination of questionnaire-based examination, skin prick test, and serum omega-5 gliadin-specific IgE test).^39^

Non-celiac gluten sensitivity (NCGS) is a recently proposed, increasingly recognized clinical condition in patients in whom celiac disease and wheat allergy have been ruled out. It is characterized by intestinal and extra-intestinal symptoms triggered by the ingestion of gluten-containing foods.^40^ Due to the lack of a reliable biomarker, confirmation of an NCGS diagnosis relies only on a double-blind placebo-controlled (DBPC) gluten challenge.^41^

So far, a gluten-free diet is the only safe and effective treatment for the above conditions of gluten intolerance.^32^

### Gluten-free “lifestylers”

Demand for gluten-free foods is not limited to the gluten-intolerant population. Although it is not clear whether a gluten-free diet is beneficial for one’s health, some gluten-tolerant consumers believe that gluten-free food products are simply healthier.^42,43^ This can be partly explained by a kind of “health halo” effect, making consumers believe that products with “free-from” label are healthier overall.^44^ Besides, some popular books by bestseller authors, athletes, and celebrities have encouraged a gluten-free diet. An online questionnaire survey demonstrated that 41% of non-celiac athletes, including Olympic medalists, follow a gluten-free diet 50–100% of the time, and that adoption of the diet in most cases was not based on a medical rationale and may have been driven by the perception that gluten removal provides health benefits and an ergogenic edge.^45^ Approximately 13% of young adults are reported to value gluten-free food; this population is more likely to engage in other healthy dietary behaviors, such as eating breakfast daily and eating more fruits/vegetables while simultaneously pursuing questionable behaviors, such as using diet pills to control weight.^42^

A double-blind randomized study found that the supposed health benefit of a gluten-free diet has no evidence base in individuals who do not have celiac disease or irritable bowel syndrome, demonstrating that gluten is unlikely to be the culprit for gastrointestinal symptoms or fatigue in otherwise healthy individuals.^43^ Moreover, commercially available gluten-free food products tend to contain ingredients with less diversity and less nutritional quality compared with their gluten-containing counterparts.^46,47^ Other studies claim that despite recent improvements in the formulation and availability of gluten-free foods, they still are less available and more expensive than gluten-containing versions.^48^ They generally have adequate levels of fiber and sugar, but lower levels of protein and higher levels of fat compared with their gluten-containing counterparts.^48^ Also, very few gluten-free foods are fortified with micronutrients.^48^

The gluten-free products market was valued at USD 4.18 billion in 2017 and this is projected to reach USD 6.47 billion by 2023, at a compound average growth rate of 7.6% during the forecast period.^49^ The gluten-free diet has become the mainstream rather than just supporting a niche market.

## Developments of gluten-free breads

As mentioned in the previous sections, demand for the development of gluten-free foods is growing.^50^ Much of the focus is on bread products, as bread is an important staple food. Rice is considered a suitable substitute for wheat, as it is available worldwide and is less allergenic. So, development of rice-based gluten-free breads is the main topic of this review. It is not easy to make bread without using wheat flour or gluten, as bread’s quality depends on the properties and functionality of gluten.^25^ In a wheat flour dough, the gluten matrix, composed mainly of the protein network of gluten, starch granules, and water (Fig. 1a), encloses the fermentation gas, making small “balloons”. Thus, the dough rises as the fermentation proceeds. On the other hand, hydration of flour from gluten-free cereals, such as rice, results in a runny “batter” rather than viscoelastic “dough” as their proteins do not possess the network-forming properties typically found in gluten.^51^ Therefore, the fermentation gases rise to the surface while starch granules and yeast settle.^52^ Generally, a gluten-free batter without a thickening agent, such as hydrocolloids, becomes foamy.^53,54^

### Additives

Several efforts have been made in the development of gluten-free breads. Typical gluten-free breads contain hydrocolloids (e.g., xanthan gum, guar gum, etc.) which increase the viscosity of the liquid phase, keeping the starch granules, yeast, and gas bubbles suspended in the fermentation process.^52,55^ The subsequent baking process gelatinizes the starch and hardens around the hydrocolloid membrane surrounding the air bubbles to set the crumb structure. As a surface-active hydrocolloid, hydroxypropyl methylcellulose (HPMC) behaves somewhat differently. It has hydrophobic methyl ester/hydroxypropyl groups in addition to hydrophilic cellulose regions. Thus, HPMC stays at the gas/liquid interface, uniquely stabilizing the bubbles and preventing coalescence.^52,56^ Moreover, as HPMC is thermoreversible,^57^ it also helps harden the bubble membrane in the baking process.^58^

Another recent approach includes enzymatic treatment of gluten-free batter.^51^ Transglutaminase (EC 2.3.2.13) catalyzes the acyl-transfer reaction between primary amino groups on protein-bound lysine residues and γ-carboxyamide groups on protein-bound glutamine residues.^59^ Thus, transglutaminase is capable of introducing covalent cross-links between proteins.^60^ The protein cross-linking ability has been shown to transform weak gluten into a strong gluten, with measurable effects on rheological behavior.^61^ The addition of transglutaminase, along with HPMC, to a gluten-free rice batter resulted in its improved elastic and viscous behavior, as well as a higher specific volume and softer crumbs in the resulting bread.^62^ The improvement in the viscoelastic properties of the rice batter appeared to be associated with the enhanced capability of the rice flour to retain the carbon dioxide produced during proofing. The quantitative decrease of free amino groups of proteins suggested that this improvement was due to the cross-linking of protein, that is, the generation of a gluten substitute, supplementing the role of HPMC in the baking of rice bread.^62^ Microstructure analyses of a rice-based bread fortified with skim milk or egg powder using confocal laser-scanning microscopy (CLSM) verified that addition of transglutaminase promoted the formation of a protein network in the gluten-free bread that mimicked the gluten network in wheat breads.^63^ The networking efficiency of transglutaminase depends on both the correct protein substrates and the level of enzyme addition. Thus, formation of the appropriate protein network under the right conditions should improve the overall quality of gluten-free bread by enhancing loaf volume and crumb characteristics, as well as appearance.

Improvement of the gas-retaining capability of gluten-free batter using protease, a seemingly paradoxical strategy for cross-linking, is also in progress. Protease has actually been used to weaken wheat dough by cleaving a portion of the gluten network.^64^ However, treatment of a brown rice batter with bacterial protease improved bread quality by significantly increasing the specific volume while decreasing crumb hardness and chewiness.^65^ CLSM images of the bread crumbs suggested that the gelatinized starch phase was the main structure component in the protease-treated bread. Thus, protease may partially degrade the large macromolecular protein complex embedding starch granules,^66,67^ resulting in improved continuity of the starch phase as well as better rheological properties of the batter. Treatment of rice batter with a protease from *Aspergillus oryzae* increased its viscosity and resulted in bread with a high specific volume. Optical microscopic observation of the batter suggested that partially degraded protein, possibly glutelin, and starch granules formed aggregations containing voids.^54^ This fine network of interlinked protein‒starch aggregates resulted in gas cell stabilization.^54^ Proteases are mainly categorized into four classes based on the catalytic mechanism: metallo, serine, cysteine, and aspartyl proteases.^68^ Comparative analyses of the proteases^69,70^ demonstrated that metallo, serine, and cysteine proteases, but not aspartyl protease, are effective additives for improving the quality of gluten-free rice breads.

### Application of the redox regulation

Addition of glutathione, a ubiquitous natural peptide, facilitated the deformation of rice batter, thus increasing its elasticity in the early stages of bread baking and increasing the volume of the resulting bread.^53,71^ Below, we would like to introduce briefly how glutathione can be used in making gluten-free rice bread. The disulfide bond is a cross-link between two cysteine residues and plays an important role in the structure/function of proteins.^72^ Redox regulation, control of reduction/oxidation of the disulfide bonds, as well as phosphorylation are the two major post-translational modifications of proteins.^73^ Thioredoxin (Trx),^74^ a small 12 -kDa protein, and glutathione,^75^ a natural tripeptide, play central roles in the redox-dependent regulatory mechanisms.

Trx reduces the disulfide bond of its target protein specifically. In the reactions below, oxidative status is abbreviated as “OX” and reduced status is abbreviated as “RED”:

2 $$\begin{array}{l}{\mathrm{Trx}}_{{\mathrm{RED}}}\left( { - {\mathrm{SH, HS}} - } \right) + {\mathrm{Target}}_{{\mathrm{OX}}}\left( { - {\mathrm{S}} - {\mathrm{S}} - } \right) \to \\ {\mathrm{Trx}}_{{\mathrm{OX}}}\left( { - {\mathrm{S}} - {\mathrm{S}} - } \right) + {\mathrm{Target}}_{{\mathrm{RED}}}\left( { - {\mathrm{SH, HS}} - } \right)\end{array}$$

Glutathione (GSH) is a tripeptide with a free SH group. Two molecules of glutathione occasionally cross-link with an intermolecular disulfide bond to make “oxidized” glutathione (GSSG). Glutathione’s reaction occasionally entails glutathionylation (GL):^76^

3 $$\begin{array}{l}{\mathrm{Target}}_{{\mathrm{OX}}}\left( { - {\mathrm{S}} - {\mathrm{S}} - } \right) + {\mathrm{G}} - {\mathrm{SH}} \to \\ {\mathrm{Target}}_{{\mathrm{GL}}}\left( { - {\mathrm{S}} - {\mathrm{SG}}, {\mathrm{HS}} - } \right)\end{array}$$

4 $$\begin{array}{l}{\mathrm{Target}}_{{\mathrm{RED}}}\left( { - {\mathrm{SH}}} \right) + {\mathrm{GS}} - {\mathrm{SG}} \to \\ {\mathrm{Target}}_{{\mathrm{GL}}}\left( { - {\mathrm{S}} - {\mathrm{SG}}} \right) + {\mathrm{GSH}}\end{array}$$

5 $${\mathrm{Target}}_{{\mathrm{OX}}}\left( { - {\mathrm{S}} - {\mathrm{S}} - } \right) + {\mathrm{GS}} - {\mathrm{SG}} \to {\mathrm{Target}}_{{\mathrm{GL}}}\left( { - {\mathrm{S}} - {\mathrm{SG}}} \right) \times 2$$

Redox regulation has been a key target of Dr. Bob Buchanan’s laboratory, University of California, Berkeley, after he clarified the Trx-dependent regulatory mechanism in photosynthesis.^77,78^ In the proteomic analyses of plant biochemistry mostly performed by the Berkeley group,^79–82^ we have found that redox regulation occurs in many aspects of plant life and plays critical roles in plant biology: seed germination/maturation, photosynthesis, defense against oxidative stress/pathogens, and others.^83^ Then, thinking in the opposite direction, modification of the disulfide bonds in biology, that is, artificial activation of the redox regulatory mechanism, might lead to the production of a new, useful plant. Following this hypothesis, overexpression of Trx in plants was first tried in the starchy endosperm of barley.^84^ The transformant germinated earlier than the wild type. Also, enzymes in charge of starch mobilization appeared earlier. As fast germination of barley seeds reduces the production cost and improves the quality of beer,^85^ the results suggest the practical utility of Trx transformants. Conversely, underexpression of Trx in white wheat seed has been tried. White wheat has received increasing attention, as it is naturally white and needs no bleaching for uses, such as breadmaking. However, white wheat grains tend to germinate on the spike before harvest.^86^ The preharvest sprouting (PHS) reduces the crop yield as well as the quality of the seeds and the flour. Rainfall or high humidity in the grain-filling season leads to PHS, and causes farmers significant financial losses.^87^ Suppression of Trx in the starchy endosperm led to improved PHS resistance in the transformants^88^ without affecting the crop yield or flour quality.^89^

These two findings reported by the Berkeley group are the first discovery that control of Trx expression, that is, artificial redox regulation, affects the physiological processes of plants. Although risk assessment of genetically modified organisms (GMOs) is a critical issue,^90^ the characteristics of these and other trial model plants provide the possibility of the industrial application of redox regulation.^91^

More recently, we have sought to use this strategy to enable rice batter to confine fermentation gas. Glutathione was added to rice batter in an attempt to transform the intramolecular disulfide bonds of rice proteins into intermolecular disulfide bonds and eventually form a gluten-like network. Both reduced glutathione (GSH) and oxidized glutathione (GSSG) were found to be successful in swelling gluten-free rice batter and bread.^53,71^ However, contrary to our expectations, analysis of the proteins revealed that no gluten-like protein network was formed. In contrast, microstructure and biochemical analyses suggested that glutathione cleaved the disulfide-linked glutelin polymers embedding the starch granules. The glutelin polymer has been suggested to work as a hindrance to the absorption of water by starch molecules when water is added to a rice flour;^66^ glutathione may fray this barrier to make the batter more consistent and viscous, thereby improving its gas-holding capability in the fermentation process,^53^ as is the case with protease-treated rice batter.^65^ Although the number of its applications in food processing has been limited so far,^91^ glutathione appears to be a promising tool for developing food with new properties. Glutathione is usable as a food ingredient in the United States^92^ and some east Asian countries. For example, glutathione-based oral dietary supplements have been accorded the status of a Generally Recognized as Safe (GRAS) constituent with Section 201(s) of the Federal Food, Drug, and Cosmetic Act of the US Food and Drug Administration (US-FDA).^93^

On the other hand, usage of glutathione for food has some limitations. First, glutathione is not usable as a food in all countries. In Japan, for instance, it is recognized as medicine, and cannot be incorporated as a food additive.^94^ Second, GSH-added rice batter has been shown to yield a slight amount of hydrogen sulfide (0.43 ppm) and methyl mercaptan (0.106 ppm) in the headspace gas of the bread.^71^ Generation of hydrogen sulfide in heated meat or purified GSH is well known;^95^ indeed, a slight amount of hydrogen sulfide contributes to the pleasant aroma of cooked meat^96^ and rice.^97^ Usage of GSSG in breadmaking instead of GSH significantly reduced the generation of these sulfur compounds,^71^ and sensory evaluation demonstrated that the aroma of GSSG-added rice bread was almost equivalent to that of non-added bread.^98^ However, we sought to develop rice bread without glutathione or any other additives.

In the process of developing glutathione-added rice bread, we found that the control sample, that is, “non-added bread”, occasionally swelled in fermentation. Although it collapsed mostly in the following baking process, we expected that if optimal conditions could be found, we could make an additive-free, gluten-free rice bread from solely the basic ingredients: rice flour, water, yeast, sugar, salt, and oil.

### Additive-free, gluten-free rice bread

The development of additive-free, gluten-free rice bread has taken a trial-and-error rather than a strategic approach.^99,100^ First, we tried several commercially available rice flours and found that flours with low-starch damage (<5%) were the most suitable. The physical property of the gluten-free rice batter appeared quite different from the familiar viscoelastic wheat dough. It had an appearance and texture of a slurry with low viscosity. So, lots of “cooking tips” have been discerned for the breadmaking process. For example, as rice batter tends to make lumps, we paid attention in the mixing procedure to avoid lumps. Also, the dried yeast needs to be dissolved completely. Generation of bubbles of different sizes due to heterogeneous distribution of dried yeast may result in their coalescence^101^ and a sudden shrinkage of the batter in the fermentation process. The breadmaking processes, i.e., mixing of the batter, fermentation and baking, as well as tips for successful making in the respective processes, are mentioned in a later section.

**Fig. 2:**
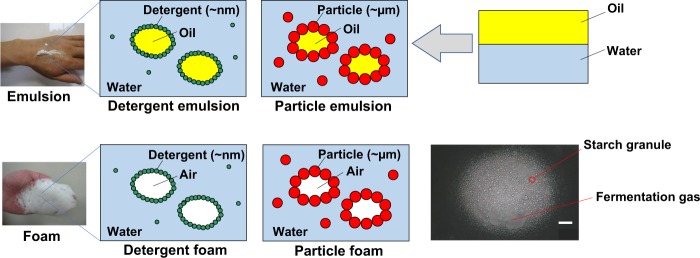
Explanatory figure of particle emulsion/foam. Adapted from refs. ^99,100^. Scale bar: 30 µm. Copyright (2017), with permission from Elsevier

To clarify how the gluten-free batter swells without additives, we sought to investigate the microstructure of the fermenting batter. The fermenting batter appeared like a meringue and was quite different from wheat dough, which is so viscoelastic that its full mass can be lifted with a scoop (Fig. 1b). As it was not easy to freeze the fragile batter without destroying the delicate structure, a sectioned specimen for microscope observation could not be made. Instead, freshly made batter was sandwiched between a microscope slide and a coverslip and the batter was left at room temperature to ferment there. Optical microscopic observation revealed the microstructure: bubbles covered by starch granules (Fig. 2). The structure was entirely different from that of the typical wheat dough, in which gas cells are surrounded by the gluten matrix made by a network of gluten protein and starch granules.^102^ In contrast, it had a similar structure to a “particle emulsion”^101^ in which rice granules stabilize the interface between oil and water (Fig. 2).^103^ Thus, it was suggested that the bubble observed in an additive-free, gluten-free rice batter had the structure of a “particle foam” (Figs. 1a, 2).^101^

The hypothetical mechanism is illustrated in Fig. 2. Generally, oil and water do not mix. However, when they are mixed well in the presence of a detergent, microscopic oil droplets covered by detergent molecules disperse throughout water. This is a classic emulsion. Likewise, aeration of water in the presence of detergent results in a foam. A small amount of air is surrounded by a thin film of water, in which detergent molecules stabilize the boundary.

At the beginning of the 20th century, solid particles were found able to adsorb onto the interface between oil and water, and play a similar role to that of detergent molecules.^104,105^ This is called a “particle-stabilized emulsion” or “particle emulsion”. Starch granules of native rice, maize, wheat,^103^ quinoa,^106^ high-pressure treated corn starch granules,^107^ chemically modified waxy maize and tapioca,^108^ as well as rice starch granules^109^ have been reported to form particle emulsions. A particle-stabilized foam occurs in the same manner. Particle emulsions/foams have received renewed attention during the past decade, as recent advancement in nanoparticle technology accelerates research trends.^110,111^ Moreover, such foams have potential applications in a wide variety of industries, including foods, pharmaceuticals, and cosmetics. One of the key advantages of the mechanism for foodstuff applications is that microparticles of biological origin, such as starch granules, cellulose, or protein particles, work as stabilizers.^101^ Our report showed for the first time that rice starch granules stabilize particle “foam” in an additive-free, gluten-free rice batter.^99^

**Fig. 3:**
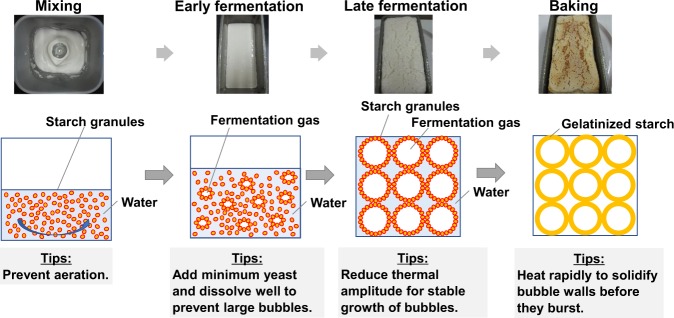
Summary of the procedures for making additive-free rice bread and “cooking tips” for each step. Adapted from ref.,^100^ with permission

The breadmaking processes and tips for the successful gluten-free breadmaking from rice flour are summarized in Fig. 3. In the early stage of fermentation, yeast produces fermentation gas, composed mainly of carbon dioxide and alcohol. Ordinarily, the batter cannot hold the gas and becomes foamy.^53,54^ However, if rice flour with low-starch damage is used and breadmaking is performed with the right conditions, the fermentation gas is trapped in the batter.^99^ Thus, small bubbles appear throughout the batter. The small bubbles are particle foams in which fermentation gas is surrounded by starch granules. As the fermentation proceeds, the fragile bubbles gradually grow bigger, making the whole batter rise. Here, it is critical to keep the temperature stable, as fragile bubbles tend to burst in fluctuating temperatures. In the late stage of fermentation, the swollen bubbles should be heated rapidly to make the starch granules gelatinize, that is, to solidify the bubble walls. The most swollen bubbles are the most fragile, so rapid heating is the key.

The overall process resembles the synthesis of a polyacrylamide hydrogel, in which modified nanoparticles stabilize an air/water (acrylamide solution) emulsion, and the macroporous structure is fixed by thermal-induced polymerization.^112^

We have investigated several commercially available rice flours and found that rice flours with less starch damage (<5%) make bread with a higher specific volume.^99^ Higher starch damage tends to facilitate greater absorption of water by starch granules.^113^ The hydrophobicity/hydrophilicity ratio determines the aptitude of starch granules to form particle foam.^114^ Thus, to prevent destabilization of the fragile bubbles in the fermentation process, it is important to maintain the proper hydrophobicity/ hydrophilicity ratio. Our success in making bread using flour with less starch damage, that is, less water absorption, seems consistent with the hypothetical mechanism. In this context, reduction of surface tension by hydrophobic treatment of rice starch granules was successful in making a stable particle emulsion.^108,109^

From another point of view, if rice starch granules are capable of constituting a particle foam, they should have the ability to mimic the function of detergents, that is, to reduce the surface tension of water. Starch granules with less starch damage (4.7 w/w%) effectively reduced the surface tension of water from 73 to 35 mN/m. In contrast, starch granules with higher starch damage (9.8 w/w%) were not as effective, reducing the surface tension to only 47 mN/m.^99^

**Fig. 4:**
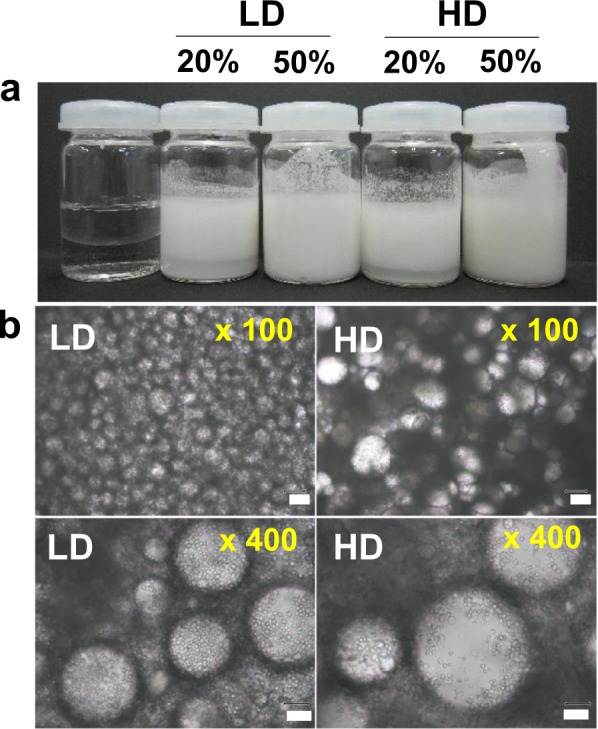
**a** Water/tetradecane emulsions formed by starch granules at different rice flour concentrations. From left to right: control (no flour), addition of rice flour with low-starch damage (20% w/w, 50% w/w), as well as high-starch damage (20% w/w, 50% w/w). **b** Optical microscopic analyses of the emulsion. Rice flour with low- (LD) and high- (HD) starch damage was compared. Adapted from ref. ^99^ Scale bar: 100 µm for ×100, and 30 µm for ×400, respectively. Copyright (2017), with permission from Elsevier

Starch granules show emulsion-forming ability by stabilizing the water/tetradecane interface.^108^ So, similar experiments were conducted using starch granules with low- and high-starch damage (Fig. 4). Both starch granules made stable water/tetradecane emulsions (Fig. 4a). However, the microstructures of the emulsions were somewhat different (Fig. 4b). Optical microscopic analyses of the emulsions showed that starch granules with less starch damage (LD) covered the oil droplets densely. In contrast, in the case of rice granules with higher starch damage (HD), swollen granules were occasionally seen, and the oil droplets were not covered completely. Thus, rice granules with low-starch damage demonstrated better particle-emulsion-forming ability compared with the high-starch-damage counterparts. This was consistent with the observation that rice starch granules with low-starch damage were suitable for constructing particle foam, that is, to make additive-free rice bread.

All these three observations support the hypothetical particle foam theory. Verification studies are in progress in our lab.

## Conclusion

Several approaches in the development of gluten-free bread by our own laboratory and others have been introduced in this review, together with the social and scientific context of these efforts. The research is aimed to improve the quality of life of celiac disease or wheat allergy patients. Better bread quality (flavor, texture, and volume), reduced production cost, and wider availability are all important issues.^115^ For example, so far, rice bread lacks the mouth-watering aroma of freshly baked wheat bread. It is not clear whether this is inevitable or whether a better selection of ingredients or an improved breadmaking procedure could lead to improvement of the aroma and flavor of rice bread, such that it becomes comparable with that of wheat bread. Besides, rice breads tend to be sticky compared with wheat bread. Also, gelatinized rice starch tends to retrograde faster,^116^ so the bread is prone to become stale and hardened faster,^117^ resulting in a shorter shelf life.^118^ Using rice varieties with intermediate amylose content and a low water absorption index may give superior crumb properties.^119^

Recent wide availability of household breadmaking countertop appliances has prompted our laboratory and others to develop gluten-free bread recipes suitable for these machines. Providing specific ingredients, such as fitted rice flour sold along with the breadmaker, may help consumers experience success in making custom gluten-free bread at home. Improving the machines by incorporating an induction-heating (IH) system may be suitable for making “particle-foam” type rice bread, as an IH system guarantees stable temperature control in fermentation as well as rapid heating in the baking process.^120^ Addition of micronutrients and functional food ingredients is also an important theme. Further studies may thus improve the bread quality to be comparable to that of wheat bread and to improve the quality of wheat-sensitive patients’ life through providing a satisfactory diet.
